# Aristocracy of Professions

**Published:** 1882-06

**Authors:** J. A. Robinson


### ARISTOCRACY OF PROFESSIONS.

BY J. A. ROBINSON, D.D.S.

Ever since we have had any history of man, there is evidence of a desire of the strong to exercise authority over the weak, and it is as distinct a characteristic as is exhibited among the lower orders of animal creation. When civilization raises us above the force of arms or mere brute force, this authority or power is gained by intrigue, assumption, and deception. Aristocracy of learning, combined with religious prejudice, has played a most important part in the subjugation of the race. The imaginary virtue of various devices—images or figures, or the pretentions of astrologers in foretelling future events by the stars, has lent such a charm to the few, that the many have been made subservient by a deception that seemed almost miraculous over the masses, and enforced an obedience that was almost absolute. We find this in the church by singing Latin mass, and mumbling of prayers that the common people cannot understand. The Latin prescription of the physician is the remains of this same endeavor to blind the eyes of the ignorant. Priestly robes, costly sacrifices, and the burning of incense, and sacred perfumes, are all a parcel and part of the plan to inspire *awe*, and so gain control of the masses by preying upon their credulity.

The unfraternal feeling that is exhibited between members of the dental profession, grows out of aristocracy. The coldness that is manifested towards the new practitioner who has just graduated, is an injury to both those who are beginning, and also to those who are well established. The really great are never pretentious. The days of secrecy and privacy ought to be abolished. The old saying of Daniel Webster, that "there is always room at the top," ought to be sounded in the ears of the old, to incite them to endeavor; and of the young to stimulate them to gain knowledge. Frederic Douglass once said that "he had never been in the presence of but one white man in his whole life for fifteen minutes but what he had been reminded, in some way, that he was a black man; and that man was Abraham Lincoln." This was because Mr. Lincoln was *really great.* Now, while we believe it is all important that professional men should be thoroughly informed in regard to their calling—should be required to pass a thorough examination in all that pertains to what is expected of them by the community, and a law passed requiring them to do so; when that is performed, they should receive the hearty countenance and endorsement by those who are *elders* in the profession, and should be regarded as friends, and not looked upon as enemies. It was the coldness of society that made such a character as Jesse James. To be sure, James was a bad man; he had committed a grievous crime, but the moment he was *outlawed* he became desperate. It is so, in some measure, with the young practitioner in any profession. Fraternal feeling—and it might go as far as "brotherly love"—ought to be extended to every one who has complied with the requirements of professional duties. Estrangements in professions on the one part produces a reflex action; and our whole profession, instead of being what it should be—that is, made up of active friendships, is as frozen as the atmosphere at the north pole.

The idea that it is unprofessional to advertise our calling is another relic of aristocracy that ought to be destroyed. The people of America get information through newspapers. They are a people who read, and, in a good many things, think. Now, will you allow those who are aliens, and we might say *enemies*, to do all the advertising, and so mislead the public, or will you allow the people to be well informed on all dental subjects by those who are competent to do that, and so prevent the wholesale extraction of teeth by those who go about "yanking" out teeth, as some do at this time?

It is the *people* that need to be informed; and while the various monthlies are teeming with dental literature and *advertisements* of the improvements in methods of work, and dental appliances; and what the dentist ought to do to obtain the best results; is it any worse to say to the world what they ought to demand of every person who makes any pretentions to perform dental operations, and would it not drive the ghosts that infest us—and, like all ghosts that disturb only the ignorant—out of the profession, and so benefit the dentist by spreading light all over the world. It is no reason why the press should not be an instrument for *good*, because the charlatan uses it as an instrument for evil

The cruelty inflicted upon community by those who extract teeth, ought to receive special attention, and the entire profession ought to be allowed to tell their neighbors of the great crime of tooth extraction, and to enforce it in such a way that the world would realize it. It should be written in every school book, and preached from every pulpit in the land. This can never be done while the profession stands so coldly apart. This fraternal feeling, this fellowship of souls we advocate, would produce harmony of action, and the people would be saved. Our whole civilization is changed, and we must change with it.

Look at the way goods are sold, and information on all subjects gained to day. Look at the means of information and education; the methods of travel, and of talking on the wings of the wind. Why, we can already almost hear the breezes singing on the top of the Rocky Mountains, and hear the surging of the waves of the ocean shores of the Pacific. Shall we shut our eyes in aristocratic night, and hold our tongues in aristocratic silence because it is unprofessional to say to the world that it is a crime to extract teeth, or to say so in a newspaper, as well as in a magazine, that is never seen or read by any but those engaged in the profession. Or shall we be allowed, by the use of printers' ink, to let the world know what we are about. Let the world know we make it a specialty to *save teeth*, and do not extract them, which is true. Let us throw away the forceps, and make the world believe they should be put aside with the lancet, and only to be used on extraordinary occasions. Let this doctrine be taught in all the schools, and let us hold up the hands of all young practitioners who will try to do this, and the profession and the world will be saved.

The improved surgery is not to amputate, but to save the organs nature has bestowed upon us, and the dental profession will be forced sooner or later to adopt the practice of the coming men, or be pronounced unworthy of the new civilization. These thoughts come from a man who has worked hard and long in the most beautiful and useful of all the professions, and from one who has never advertised, or had a sign on his office for the past five years, but one who writes what he believes to be the new truth of the age in which we live.

